# Gaps in universal health coverage in South Korea: Association with depression onset in a community cohort

**DOI:** 10.1371/journal.pone.0197679

**Published:** 2018-06-11

**Authors:** Hye Yin Park, Yun-Chul Hong, Ichiro Kawachi, Juhwan Oh

**Affiliations:** 1 Institute of Environmental Medicine, Seoul National University College of Medicine, Seoul, Republic of Korea; 2 Department of Preventive Medicine, Seoul National University College of Medicine, Seoul, Republic of Korea; 3 Department of Social and Behavioral Science, Harvard T.H.Chan School of Public Health, Boston, MA, United States of America; 4 JW LEE Center for Global Medicine, Seoul National University College of Medicine, Seoul, Republic of Korea; Griffith University - Gold Coast Campus, AUSTRALIA

## Abstract

**Background:**

While controversies on limitation of coverage by the national health insurance and relatively high direct or “out-of-pocket” household payments by the national health insurance in South Korea still remain, potential unfavorable influence of the insufficiency of the universal health coverage on depression has not yet been evaluated.

**Methods and findings:**

Baseline information were obtained from a community cohort (The Korean Genome and Epidemiology Study) of middle-aged subjects without depression at enrollment period (2001–2002). Subjects were followed-up biennially, and new onset depression was assessed using Becks Depression Inventory at 2nd round follow-up (2005–2006). Influence of direct medical expenditure on depression onset was investigated in all subjects and in stratified groups of different income level. Increasing risk of depression onset was observed for increased medical expenditure (OR [95% CI];1.44 [0.97–2.13], 1.90 [1.19–3.05], 1.71 [1.01–2.91] for spending <50000 KRW, 50000–100000 KRW, and ≥100000 KRW, respectively, vs. almost no expenditure per month; P for trend = 0.012), after adjusting for covariates such as monthly income and chronic disease history. Similar associations were observed in subjects less than or at average national income, but results were not significant in subgroup with monthly income above national average.

**Conclusions:**

Even with the universal coverage, high co-payments and uninsured services in the Korean health insurance system yet possibly make the insured pay much for medical service utilization. This might have led to onset of an unfavorable health condition such as depression.

## Introduction

Since the full-population coverage extension of the national health insurance (NHI) in 1989, South Korea has observed gradual increase in volume of covered services as well as decrease in out-of-pocket household expenditure on purchasing health care [1]. While the extended NHI with relatively low service price has lowered the burden on household medical expenses, arguments on limited range of benefit coverage and relatively high direct (out-of-pocket) payments by households still remain [1,2]. Moreover, not only would the low coverage and low level of fee schedule lead to increased direct household payments but also to distorted medical practice patterns as well as continuous expansion of non-covered services, and would eventually lower satisfaction of the insured population [3,4].

Insufficient coverage may force people to struggle with either unmet need or financial burden for their met need. Of these two unfavorable circumstances, attempts have been made in answering the question on if unmet health care need lead to unfavorable final outcome in terms of health status. A cohort study of 1,315 noninstitutionalized elderly subjects in Barcelona, Spain reported that group with unmet health care need showed higher risk of mortality [5]. More recently, a 4-year panel study in South Korea, which investigated association of unmet health care need with health outcome measured by health-related quality of life instrument (EQ-5D) and self-rated health, also found an inverse relationship between unmet health care need and the health outcome indices [6]. A cross-sectional study in Japan, which used regression discontinuity model, showed mental and physical health gain in the group eligible for the Japan’s reduction in health care cost-sharing [7], and a community-level intervention on insurance benefits in USA also resulted in higher healthcare utilization, lower out-of-pocket medical expenditure as well as better subjective physical and mental health [8]. However, the role of direct household medical expense on health outcome in terms of disease incidence has not yet been evaluated based on longitudinal study setting.

Thus, the aim of this study was to examine whether direct medical service payment burden influences depression onset in an urban cohort population.

## Methods

### Study population

Study subjects consisted of 3,423 persons (who had no depression at the baseline period (2001–2002) and completed follow-up over time by the 2^nd^ round follow-up period (2005–2006) with medical payment data) from the participants of Ansan-Aseong Cohort Study, an ongoing community-based cohort that is part of the Korean Genome and Epidemiology Study (KoGES) [9]. Of the cohort population, Anseong participant were excluded since depression was not evaluated during follow-up period in this population. Ansan (urban area south of Seoul) Cohort, consisting 5,020 residents aged 40–69 years at baseline, was utilized for this study as they had their depression symptoms checked at the second round. After enrollment in the years 2001–2002, participants have been followed up biennially. Baseline information on disease history, socioeconomic status, lifestyle habits, etc. were obtained through self-report questionnaires, and physical examinations were also carried out. Among 5,020 Ansan participants, 30 peoples having history of psychiatric disease at the baseline (2001–2002) and 1,567 subjects who were not evaluated for depression severity at 2^nd^ follow-up were excluded, yielding 3,423 participants in final. The study population with 3,423 were followed up for depression onset for four years by the year 2005–2006.

### Study variables

#### Outcome variable (depression)

Beck’s Depression Inventory (BDI-II) is a validated 21-questions self-report inventory for measuring depression severity [10], with value of 0 to 3 scores assigned for each question. The cutoffs of BDI-II are as follows: 0–10, normal; 11–16, mild mood disturbance; 17–20, borderline clinical depression; 21–30, moderate depression; 31–40, severe depression; and >40, extreme depression. In this study, a cut-off score of 0–20 was used to make a binary outcome variable, i.e. BDI-II scores <21 as absence of depression (i.e. depression intensity is negligible in terms of clinical treatment), and ≥21 as presence of ‘clinically considerable’ depression.

#### Independent variable (medical expense)

Medical expenses were checked according to subjects’ answers to the question “How much is your average monthly medical expenses?”, with answer choices categorized as ‘almost none’, ‘<50,000 KRW’, ‘50,000–100,000 KRW’, or ‘≥ 100,000 KRW’ (10,000 KRW approximately equaled to 12.5 USD in 2001–2002).

#### Co-variates

Age was recorded as continuous variable, and gender as a binary variable (‘male’ or ‘female’). Body mass index (BMI) was calculated from measured heights and weights taken on the same day the subjects answered to questionnaires, and were grouped as ‘underweight (<18.5 kg/m^2^)’, ‘normal weight (18.5–23 kg/m^2^)’, or ‘overweight/obese (≥23 kg/m^2^)’. Subjects were asked on their final education level, and with the given information we grouped education attainment as ‘middle school or lower’, ‘high school’, or ‘college or more’. Subjects also answered for monthly household income, which was adjusted for number of household members, then categorized into three groups of ‘below minimum cost of living’, ‘above minimum and at average cost of living’, and ‘above average cost of living’, with reference from the national statistics for minimum cost of living and average income per number of household (www.kosis.kr). Home ownership was categorized into three groups: “home owner”, ‘renting’ or ‘other’. Smoking status was determined from subjects’ reports of ‘never smoked’, ‘have smoked in the past but quit’, or ‘currently smoking’. Subjects were asked if they regularly exercised, and if they usually had insomnia, with choice of answers as ‘yes’ or ‘no’. They were also asked on frequency of eating alone, with answer choices such as ‘almost never’, ‘1–3 times per week’, ‘4–6 times per week’, and ‘more than 1 meal per day’.

Chronic disease conditions were collected from self-report questionnaires, and we created a categorical variable for chronic disease status at baseline. Answering “yes” per each of the 13 certain chronic-state diseases (hypertension, diabetes mellitus, myocardial ischemia, thyroid disease, congestive heart failure, coronary artery disease, asthma, chronic obstructive pulmonary disease, renal disease, hepatitis, tuberculosis, dementia, cerebrovascular disease) was treated to a value of 1, then all values were summed to produce a score ranging from 0 to 13 at maximum. This score was categorized to three subgroups: having 0, 1 or ≥2 chronic diseases.

### Statistical analysis

Test for independence between baseline characteristics of study subjects and depression were carried out using Mantel-Haenszel chi-square or Fisher’s exact test for categorical variables and t-test for continuous variables.

Multivariate logistic analysis was used to estimate the effect of selected factors on depression in odds ratios (OR) and their 95% confidence intervals (CI). A priori covariates at baseline as well as variables that showed statistical significance in tests for independence were tested in a univariate regression model at significant level of P≤ 0.2, then the variables were fitted in a multivariate model with selection of main-effects model variables at P≤ 0.1. Likelihood ratio was tested throughout the stepwise selection procedures, as well as tests for multicollinearity. For comparing models with and without variables of interest, we tested for model discrimination using c-statistic. Association analyses were also carried out in stratified groups of different income level, i.e. in subgroup with monthly income below or at national average, versus subgroup with monthly income above national average.

In sensitivity analysis, we further excluded 137 subjects who reported to be insured under Medicaid (119 subjects), not have any public insurance coverage (14), or did not answer (4), respectively, and the association analyses were tested in 3,286 subjects with national health insurance only.

All analyses were performed using SAS version 9.4 (SAS Institute, Cary, NC, USA).

### Ethical statement

The KoGES was approved by the institutional review board (IRB) of the Korea Centers for Disease Control and Prevention (KCDC)(9), and as this data is publicly available, the IRB of Seoul National University Hospital deemed it exempt from review.

## Results

Baseline characteristics of 3,423 study subjects and their differences by depression status at follow-up are shown in Table 1. Apart from age and sex, socioeconomic factors in significant difference by depression included education level, house ownership, and monthly income. Lifestyle factors included smoking status, physical activity, frequency of eating alone and habitual insomnia, while medical expenditure per month, history of hypertension and chronic disease, were the significant physical and mental well-being factors (P <0.05).

**Table 1:** Baseline characteristics of study subjects by incidence of depression.

		All subjects	Depression/ No depression (n)	Depression (%)	P-diff
***N (%)***					
**Gender**	**Male**	1790 (52.3)	72/1718	4.0	< .0001
	**Female**	1633 (47.7)	140/1493	8.6	
**Education attainment**	**≤Middle school**	1131 (33.1)	115/1016	10.2	< .0001
	**High school**	1528 (44.7)	76/1452	5.0	
	**≥College**	759 (22.2)	21/738	2.8	
**Home ownership**	**Home owner**	2610 (76.3)	138/2472	5.3	< .0001
	**Renting/other**	809 (23.7)	74/735	9.1	
**Income per month**	**> average**	908 (26.7)	27/881	3.0	< .0001
	**>minimum, ≤average**	2231 (65.7)	143/2088	6.4	
	**≤ minimum**	259 (7.6)	40/219	15.4	
**Health insurance type**	**None**	14 (0.4)	1/13	7.1	0.102
	**National health insurance**	3286 (96.1)	199/3087	6.1	
	**Medical care**	119 (3.5)	12/107	10.1	
**Medical expenses per month**	**Almost none**	915 (26.8)	43/872	4.7	0.004
	**≤50000**	1625 (47.6)	99/1526	6.1	
	**50000–100000**	530 (15.5)	41/489	7.7	
	**≥100000**	346 (10.1)	29/317	8.4	
**Religion**	**Yes**	2404 (70.3)	148/2256	6.2	0.869
	**No**	1015 (29.7)	64/951	6.3	
**History of diabetes mellitus**	**No**	3238 (94.6)	198/3040	6.1	0.425
	**Yes**	185 (5.4)	14/171	7.6	
**History of hypertension**	**No**	3014 (88.1)	176/2838	5.8	0.020
	**Yes**	409 (12.0)	36/373	8.8	
**History of chronic dz.**	**None**	2380 (69.5)	127/2253	5.3	< .0001
	**1**	826 (24.1)	57/769	6.9	
	**≥2**	217 (6.3)	28/189	12.9	
**Smoking**	**No**	1968 (57.6)	136/1832	6.9	0.328
	**Ex-smoker**	663 (19.4)	25/638	3.8	
	**Yes**	783 (22.9)	51/732	6.5	
**Physical exercise**	**No**	574 (16.8)	23/551	4.0	0.018
	**Yes**	2844 (83.2)	188/2656	6.6	
**Frequency of eating alone**	**Almost never **	1395 (41.3)	68/1327	4.9	0.013
	**1–3 times per week**	538 (15.9)	36/502	6.7	
	**4–6 times per week**	161 (4.8)	15/146	9.3	
	**≥ Once daily**	1288 (38.1)	92/1196	7.1	
**Regular insomnia**	**No**	2925 (85.9)	145/2780	5.0	< .0001
	**Yes**	482 (14.2)	67/415	13.9	
***Mean ± SD***					
**Age (yr)**		48.7 ± 7.5	48.4/52.4		< .0001
**Body mass index (kg/m**^**2**^**)**		24.7 ± 2.9	24.8/24.4		0.070

The results of main analyses are shown in Table 2. After model selection process, significant variables were age, sex, body mass index (BMI), education level, income per month, house ownership, smoking status, physical exercise, habits of eating alone and insomnia, medical expenditure per month, and history of chronic disease. Increasing OR was observed for increased medical expenditure (OR [95% CI];1.44 [0.97–2.13], 1.90 [1.19–3.05], 1.71 [1.01–2.91] for spending <50000 KRW, 50000–100000 KRW, and ≥100000 KRW, respectively, when compared to the group with almost no expenditure per month; P for trend = 0.01). To discern the influence of monthly medical expenditure from the influence of chronic health condition on depression, we separated the variable “history of chronic disease” (Table 2, [A]) from the full model (Table 2, [B]). The area under the curve (AUC) did not differ significantly from each other between the two models (ΔAUC = 0.004), with insignificant p-value for discrimination (P = 0.17). Test for interaction effect by multiplicative model between medical expenditure and chronic disease history was also not significant (1.03 [0.77–1.37], P = 0.86).

**Table 2:** Association between medical expense and depression onset at 4-year follow-up.

		(A) Model without chronic disease history		(B) Full model	
		Odds ratio (95% CI)	P-trend	Odds ratio (95% CI)	P-trend
**Medical expenses per month (KRW)**	**almost none**	Ref.	0.009	Ref.	0.012
	**<50,000**	1.47 (0.996–2.16)		1.44 (0.97–2.13)	
	**50,000–100,000**	1.92 (1.20–3.07)		1.90 (1.19–3.05)	
	**≥100,000**	1.76 (1.04–2.97)		1.71 (1.01–2.91)	
**Age (yr)**		1.04 (1.02–1.06)		1.03 (1.01–1.06)	
**Gender**	**Male**	Ref.		Ref.	
	**Female**	3.34 (1.96–5.68)		3.28 (1.93–5.60)	
**Body mass index (kg/m**^**2**^**)**	**18.5–23**	Ref.	0.023	Ref.	0.017
	**<18.5**	2.41 (0.85–6.84)		2.53 (0.89–7.18)	
	**≥23**	0.77 (0.56–1.06)		0.76 (0.55–1.04)	
**Education attainment**	**≥college**	Ref.	0.003	Ref.	0.002
	**high school**	1.50 (0.90–2.5)		1.54 (0.93–2.57)	
	**≤middle school**	2.11 (1.25–3.56)		2.19 (1.30–3.70)	
**Income per month**	**> average**	Ref.	0.023	Ref.	0.002
	**>minimum, ≤average**	1.39 (0.89–2.17)		1.39 (0.89–2.17)	
	**≤ minimum**	2.43 (1.37–4.32)		2.46 (1.38–4.37)	
**Home ownership**	**home owner**	Ref.		Ref.	
	**renting/other**	1.52 (1.11–2.09)		1.51 (1.10–2.08)	
**Smoking**	**never**	Ref.	< .0001	Ref.	< .0001
	**ex-smoker**	1.88 (1.001–3.54)		1.88 (0.998–3.54)	
	**current smoker**	2.95 (1.71–5.08)		2.95 (1.71–5.09)	
**Regular physical exercise**	**Yes**	Ref.		Ref.	
	**No**	1.49 (0.93–2.37)		1.48 (0.93–2.36)	
**Eating alone**	**almost never**	Ref.	0.123	Ref.	0.136
	**1–3 meals per week**	1.44 (0.93–2.24)		1.43 (0.93–2.22)	
	**4–6 meals per week**	2.03 (1.09–3.78)		2.04 (1.10–3.80)	
	**≥1 meal per day**	1.32 (0.94–1.87)		1.31 (0.93–1.85)	
**Insomnia**	**No**	Ref.		Ref.	
	**Yes**	2.2 (1.58–3.07)		2.15 (1.54–3.00)	
**History of chronic disease**	**0**			Ref.	0.150
	**1**			0.97 (0.69–1.38)	
	**≥2**			1.67 (1.02–2.74)	
***AUC (95% CI)***		*0*.*751 (0*.*718–0*.*785)*		*0*.*755 (0*.*722–0*.*78)*	

Models are adjusted for variables listed in the table.

These results were robust even after adjusting number of chronic health conditions. Having had ≥2 chronic diseases at baseline compared to none had 1.67 times higher risk of depression (1.67 [1.02–2.74]).

Risk of depression in females were more than three times higher than males (3.28 [1.93–5.60]), and while the higher OR in low BMI groups (2.53 [0.89–7.18]) and lower OR in high BMI groups (0.76 [0.55–1.04]) were not statistically significant, significant linear trend was observed (P for trend = 0.02). In scope of socioeconomic status, low education level (2.19 [1.30–3.70] in subjects who finished middle school or lower compared to those with college or more), low monthly income (2.46 [1.38–4.37] in those below minimum cost of living vs. above average income), and those without self-owned house (1.51 [1.10–2.08] vs. self-owned) were significantly associated with incidence of depression at 4-year follow-up. From lifestyle factors, current smoking (2.95 [1.71–5.09] vs. never-smoked), eating alone (2.04 [1.10–3.80] in having 4–6 meals per week vs. almost never), and tendency to insomnia (2.15 [1.54–3.00] vs. no insomnia) affected depression incidence.

When stratified by monthly income level (Table 3), similar association results were observed in subjects earning less than or at average national income (OR [95% CI]; 1.39 [0.91–2.13], 2.00 [1.21–3.31], 1.70 [0.96–3.00] for spending <50000 KRW, 50000–100000 KRW, and ≥100000 KRW, respectively, compared to the group reporting almost no expenditure per month; P for trend = 0.02), but the association as well as the test for trend between medical expenditure and depression were not significant in the subgroup with monthly income above the national average (OR [95% CI]; 1.63 [0.59–4.51], 1.24 [0.27–5.63], 1.90 [0.44–8.26] for spending <50000 KRW, 50000–100000 KRW, and ≥100000 KRW, respectively, vs. almost no expenditure per month; P for trend = 0.47). In both groups, history of chronic disease did not show significant relationship with depression onset.

**Table 3:** Association between medical expense and depression onset at 4-year follow-up in different income groups.

		Subgroup with monthly income below or at average (n = 2,490)		Subgroup with monthly income above average (n = 908)	
		Odds ratio (95% CI)	P-trend	Odds ratio (95% CI)	P-trend
**Medical expenses per month (KRW)**	**almost none**	Ref.	0.015	Ref.	0.467
	**<50,000**	1.39 (0.91–2.13)		1.63 (0.59–4.51)	
	**50,000–100,000**	2.00 (1.21–3.31)		1.24 (0.27–5.63)	
	**≥100,000**	1.70 (0.96–3.00)		1.90 (0.44–8.26)	
**Age (yr)**		1.04 (1.02–1.06)		1.03 (0.97–1.11)	
**Gender**	**Male**	Ref.		Ref.	
	**Female**	2.99 (1.70–5.28)		7.21 (1.55–33.49)	
**Body mass index (kg/m**^**2**^**)**	**18.5–23**	Ref.	0.015	Ref.	0.889
	**<18.5**	2.71 (0.92–7.97)		N/A	
	**≥23**	0.74 (0.53–1.05)		0.86 (0.33–2.23)	
**Education attainment**	**≥college**	Ref.	0.006	Ref.	0.056
	**high school**	1.40 (0.79–2.51)		1.84 (0.62–5.44)	
	**≤middle school**	2.03 (1.14–3.60)		3.36 (0.96–11.77)	
**Home ownership**	**home owner**	Ref.		Ref.	
	**renting/other**	1.48 (1.06–2.08)		2.35 (0.92–6.01)	
**Smoking**	**never**	Ref.	0.0004	Ref.	0.024
	**ex-smoker**	1.83 (0.94–3.59)		2.32 (0.36–14.78)	
	**current smoker**	2.81 (1.57–5.02)		5.28 (1.14–24.53)	
**Regular physical exercise**	**Yes**	Ref.		Ref.	
	**No**	1.52 (0.92–2.51)		1.39 (0.40–4.84)	
**Eating alone**	**almost never**	Ref.	0.054	Ref.	0.448
	**1–3 meals per week**	1.48 (0.92–2.38)		1.36 (0.47–3.99)	
	**4–6 meals per week**	2.46 (1.29–4.68)		N/A	
	**≥1 meal per day**	1.44 (0.995–2.09)		0.76 (0.31–1.91)	
**Insomnia**	**No**	Ref.		Ref.	
	**Yes**	2.19 (1.53–3.13)		1.93 (0.71–5.26)	
**History of chronic disease**	**0**	Ref.	0.245	Ref.	0.226
	**1**	1.004 (0.69–1.45)		0.87 (0.30–2.52)	
	**≥2**	1.53 (0.89–2.61)		3.19 (0.89–11.36)	

Models are adjusted for variables listed in the table.

Results from the sensitivity analyses with subjects under NHIS (excluding Medicaid beneficiaries) only showed similar association as in the total subjects (S1 & S2 Tables).

## Discussion

This study showed that the people with higher out-of-pocket medical expenditure had higher risk of new onset depression over the 4^th^ year follow-up after adjusting for other known predictors of depression including monthly income and chronic health condition. This finding was robust in the subgroup of people at or below the national average income level, but not in the more affluent subgroup. The unfavorable results shed light on insufficient coverage of Korean Health Insurance Scheme in terms of all three dimension of universal health coverage. Even with 100% coverage of population dimension, high co-payments and many uninsured services in the Korean health insurance system could make people pay still a lot at the point of medical service utilization. This may, along with typical influential factors, lead to psychological distress in the middle-aged and the elderly in Korea. Provision of sufficient health insurance coverage is crucial for health sustenance in the near-elderly population as they experience increasing risk of major health problems and the consequential escalation of medical expenses [11,12]. Through longitudinal study setting, these results(more burden on out-of-pocket medical expenditure associated with more new onset depression) may enhance the level of causal inference of the previously suggested cross-sectional association (improved mental health of the target population when the Japanese government reduced the burden on out-of-pocket medical expenditures) [7].

The OECD Health Data 2009 reported South Korea to be the third-ranked country on highest “out-of-pocket” medical expenses in 2007 [13]. In 2006, the proportion of household payments over total medical expenses was 36.8%, more than twice the proportion of Japan (15.1%), another OECD country with NHI. To narrow the gap, researchers have suggested improving health care policies such as giving more weight to insurance benefits on diseases with large medical care costs, diversifying or segmentalizing fee system, and developing elderly-focused policies [3,14].

While previous studies report low income and poor insurance status as primary factors to inadequate health care in the United States [15,16], countries or regions with universal coverage also report unmet health care need [17]. A 2007–2009 Korea National Health and Nutrition Examination Survey (KNHANES) study also reported that while there was no pro-poor and pro-rich inequality in outpatient and hospital visits, it was evident that substantial out-of-pocket under the NHI were disadvantages to the poorest quintile group, especially in terms of health care quality and financial burden [18]. A recent study used 2009–2012 Korea Health Panel data with 7,717 subjects to investigate relationship of unmet health care need and EQ-5D as well as self-rated health at 1-year lag, with adjustment including insurance type and private health insurance [6]. The researcher found that 14.5% subjects reported of unmet health care need, and by regression analysis, unmet needs were associated with 1% and 4.5% decrease in EQ-5D and self-rated health, respectively. To our knowledge, however, investigation on the insufficiency of universal health coverage using new onset of illness or pre-illness as outcome measurement has not been carried out, and this is the first research to investigate such relationship using a community cohort study.

Although our study subjects were sampled from residents of a single city, our results on factors to depression selected by statistical modeling are in good agreement with previous national-level epidemiological studies in South Korea [19,20]. Our finding supports that unmet health care due to insufficient health care system may lead to unfavorable health outcomes such as depression (a mental health disorder receiving increasing attention in South Korea for its increasing, high prevalence and close relationship with suicide, another serious social issue). Also, while depressive disorder is one of the major causes of YLDs in South Korea, with 11% increase since 1990 (5^th^ in rank) to 2013, (4^th^) [21] it should be noted that the disease itself is also reported to have led to significant functional limitations and economic burden in the cohort study of near-elderly population [22]. Thus tracking depression trends in South Korea is important as it may serve as one of the health outcome indices in evaluating efficiency of health care system.

A recent study reported increased health utilization patterns in groups with new or continued Medicaid status [4], and another research also showed that being insured for Medicaid does not restrict health care utilization but even possibly leads to moral hazard [3,23]. On the other side, trend of equity on health care utilization over 1998 to 2007 in South Korea by Le Grand coefficient showed that although high utilization was observed the in low income group, increasing utilization in correlation with increasing out-of-pocket payments was conspicuous in the high income group, implying that health care utilization during the expanding coverage period was advantageous to the high income group only [24]. In our study, about 3.5% of the study population were covered by Medicaid at baseline, and subgroup analysis with elimination of Medicaid subjects produced similar results. Taken together, it is likely that the insurance type is not the core issue on health care inefficiency over the insurance system itself. Nonetheless, future investigations on number of Medicaid subjects sufficient enough for analysis is encouraged, as extended coverage in low income group was reported to lower out-of-pocket medical expenditure and—while not affecting other chronic diseases—decrease depression incidence in the Oregon experiment, a well-known randomized trial on Medicaid in United States [8,25], with which our study results aligned.

### Limitations

Although depression was evaluated using a well-validated tool, this outcome measurement is not based on a clinician assessment, nor did we use a clinical diagnostic tool (i.e. Diagnostic and Statistical Manual of Mental Disorders, DSM). Consideration is needed in interpreting the results, as depression in this study is investigated in a cohort of general population.

There was no information on purchase of private health insurance (PHI), which could alter health utilization patterns, especially in subjects with covered illnesses. However, many investigations on effect of PHI on unmet health care needs are controversial or even skeptical. Researchers reported that PHI influenced outpatient visit or expenditure and hospitalization period but inpatient visits or hospitalization, and also suggested that PHI was did not lower or even aggravated meeting unmet health care [2,26–28]. From these findings, we can surmise that reinforcing NHI guarantee takes precedence over PHI in satisfying the unmet health care. At the same time, in scope of outcome measurement, depression was not covered by private insurance systems during the study period, thus bias due to difference in health utilization patterns and the eventual disease prevention for depression by PHI is negligible.

## Conclusions

From a longitudinal study of 4-years follow-up, this study of Korean middle-aged subjects found association between increased medical expense and onset of an unfavorable health condition such as depression. These results may support every effort to make any insufficient hearth service coverage to be more comprehensive in the world including Korea.

## Supporting information

S1 Table STROBE checklist. (DOCX)

S2 Table Association between medical expense and depression onset at 4-year follow-up (NHIS subjects only: N = 3,286). (DOCX)

S3 Table Association between medical expense and depression onset at 4-year follow-up in different income groups (NHIS subjects only: N = 3,286). (DOCX)
