# Supplementary material for: Gaps in universal health coverage in South Korea: Association with depression onset in a community cohort
Source: PLoS One. 2018 Jun 11;13(6):e0197679. doi: 10.1371/journal.pone.0197679 (PMC5995437; doi:10.1371/journal.pone.0197679)
Supplement: S1 Table — (DOCX) [file pone.0197679.s001.docx]

**S1 Table. STROBE Checklist**

|  | Item No | Recommendation | Section paragraph(s) |
| --- | --- | --- | --- |
| **Title and abstract** | 1 | (*a*) Indicate the study’s design with a commonly used term in the title or the abstract.  The study design is included in the title of the paper: " Gaps in Universal Health Coverage in South Korea: Association with Depression Onset in a Community Cohort”. | - |
|  |  | (b) Provide in the abstract an informative and balanced summary of what was done and what was found.  We have provided this information in the Methods and Findings sections of our abstract:  ” Methods and Findings: Baseline information were obtained from a community cohort (The Korean Genome and Epidemiology Study) of middle-aged subjects without depression at enrollment period (2001/02). Subjects were followed-up biennially, and depression onset using Becks Depression Inventory was assessed at 2nd round follow-up (2005/06). Influence of direct medical expenditure on depression onset was investigated in all subjects and in stratified groups of different income level. Increasing risk of depression onset was observed for increased medical expenditure (OR [95% CI];1.44 [0.97-2.13], 1.90 [1.19-3.05], 1.71 [1.01-2.91] for spending <50000 KRW, 50000-100000 KRW, and ≥100000 KRW, respectively, vs. almost no expenditure per month; P for trend = 0.012), after adjusting for covariates such as monthly income and chronic disease history. Similar associations were observed in subjects less than or at average national income, but results were not significant in subgroup with monthly income above national average.” | 2 |
| Introduction | | |  |
| Background/rationale | 2 | Explain the scientific background and rationale for the investigation being reported | 1-2 |
| Objectives | 3 | State specific objectives, including any prespecified hypotheses | 3 |
| Methods | | |  |
| Study design | 4 | Present key elements of study design early in the paper | Paragraph 3 in the introduction. |
| Setting | 5 | Describe the setting, locations, and relevant dates, including periods of recruitment, exposure, follow-up, and data collection | 1-3 |
| Participants | 6 | (*a*) Give the eligibility criteria, and the sources and methods of selection of participants. Describe methods of follow-up | 1-2 |
|  |  | (*b*) For matched studies, give matching criteria and number of exposed and unexposed | N/A |
| Variables | 7 | Clearly define all outcomes, exposures, predictors, potential confounders, and effect modifiers. Give diagnostic criteria, if applicable | 2-5 |
| Data sources/ measurement | 8* | For each variable of interest, give sources of data and details of methods of assessment (measurement). Describe comparability of assessment methods if there is more than one group | 2-5 |
| Bias | 9 | Describe any efforts to address potential sources of bias | 4-5, 8 |
| Study size | 10 | Explain how the study size was arrived at | 1, 8 |
| Quantitative variables | 11 | Explain how quantitative variables were handled in the analyses. If applicable, describe which groupings were chosen and why | 2 |
| Statistical methods | 12 | (*a*) Describe all statistical methods, including those used to control for confounding | 7-9 |
|  |  | (*b*) Describe any methods used to examine subgroups and interactions | 9 |
|  |  | (*c*) Explain how missing data were addressed | 1, 9 |
|  |  | (*d*) If applicable, explain how loss to follow-up was addressed | 1, 9 |
|  |  | (*e*) Describe any sensitivity analyses | 9 |
| Results | | |  |
| Participants | 13* | (a) Report numbers of individuals at each stage of study—eg numbers potentially eligible, examined for eligibility, confirmed eligible, included in the study, completing follow-up, and analysed | 1 and Methods, paragraphs 1 and 9 |
|  |  | (b) Give reasons for non-participation at each stage | Methods, paragraphs 1 and 9 |
|  |  | (c) Consider use of a flow diagram | - |
| Descriptive data | 14* | (a) Give characteristics of study participants (eg demographic, clinical, social) and information on exposures and potential confounders | 1 |
|  |  | (b) Indicate number of participants with missing data for each variable of interest | N/A |
|  |  | (c) Summarise follow-up time (eg, average and total amount) | Methods, paragraph 1 |
| Outcome data | 15* | Report numbers of outcome events or summary measures over time | Table 1 |
| Main results | 16 | (*a*) Give unadjusted estimates and, if applicable, confounder-adjusted estimates and their precision (eg, 95% confidence interval). Make clear which confounders were adjusted for and why they were included | 2 |
|  |  | (*b*) Report category boundaries when continuous variables were categorized | Table 2 |
|  |  | (*c*) If relevant, consider translating estimates of relative risk into absolute risk for a meaningful time period | Tables 2 & 3 |
| Other analyses | 17 | Report other analyses done—eg analyses of subgroups and interactions, and sensitivity analyses | Tables S2 & S3 |
| Discussion | | |  |
| Key results | 18 | Summarise key results with reference to study objectives | 1 |
| Limitations | 19 | Discuss limitations of the study, taking into account sources of potential bias or imprecision. Discuss both direction and magnitude of any potential bias | 6 |
| Interpretation | 20 | Give a cautious overall interpretation of results considering objectives, limitations, multiplicity of analyses, results from similar studies, and other relevant evidence | 2-4 |
| Generalisability | 21 | Discuss the generalisability (external validity) of the study results | 5 |
| Other information | | |  |
| Funding | 22 | Give the source of funding and the role of the funders for the present study and, if applicable, for the original study on which the present article is based.  We have included this information in our financial disclosure:  ” Obtained funding: None.”  “Support: This study was provided with data from the Korean Genome and Epidemiology Study (4851-302) that was supported by the Korea Center for Disease Control and Prevention, Republic of Korea.” | - |

*Give information separately for exposed and unexposed groups.

**Note:** Information on the STROBE Initiative is available at http://www.strobe-statement.org.
