# Supplementary material for: Gaps in universal health coverage in South Korea: Association with depression onset in a community cohort
Source: PLoS One. 2018 Jun 11;13(6):e0197679. doi: 10.1371/journal.pone.0197679 (PMC5995437; doi:10.1371/journal.pone.0197679)
Supplement: S2 Table — (DOCX) [file pone.0197679.s002.docx]

**S2 Table. Association between medical expense and depression onset at 4-year follow-up (NHIS subjects only: N=3,286)**

|  |  | **(A) Model without chronic disease history** | | **(B) Full model** | |
| --- | --- | --- | --- | --- | --- |
|  |  | **Odds ratio (95% CI)** | **P-trend** | **Odds ratio (95% CI)** | **P-trend** |
| **Medical expenses per month (KRW)** | **almost none** | Ref. | 0.01 | Ref. | 0.02 |
|  | **<50,000** | 1.48 (0.99-2.21) |  | 1.44 (0.96-2.16) |  |
|  | **50,000-100,000** | 1.97 (1.21-3.20) |  | 1.93 (1.18-3.14) |  |
|  | **≥100,000** | 1.75 (1.02-3.00) |  | 1.69 (0.98-2.91) |  |
| **Age (yr)** |  | 1.03 (1.01-1.06) |  | 1.03 (1.01-1.05) |  |
| **Gender** | **Male** | Ref. |  | Ref. |  |
|  | **Female** | 3.28 (1.9-5.66) |  | 3.22 (1.86-5.56) |  |
| **Body mass index (kg/m^2^)** | **18.5-23** | Ref. | 0.03 | Ref. | 0.02 |
|  | **<18.5** | 2.48 (0.87-7.03) |  | 2.61 (0.92-7.40) |  |
|  | **≥23** | 0.78 (0.56-1.09) |  | 0.77 (0.55-1.07) |  |
| **Education attainment** | **≥college** | Ref. | 0.001 | Ref. | 0.001 |
|  | **high school** | 1.52 (0.9-2.56) |  | 1.56 (0.92-2.63) |  |
|  | **≤middle school** | 2.26 (1.32-3.86) |  | 2.34 (1.37-4.01) |  |
| **Income per month** | **> average** | Ref. | 0.006 | Ref. | 0.006 |
|  | **>minimum, ≤average** | 1.36 (0.87-2.12) |  | 1.36 (0.87-2.12) |  |
|  | **≤ minimum** | 2.35 (1.30-4.25) |  | 2.37 (1.31-4.29) |  |
| **Home ownership** | **home owner** | Ref. |  | Ref. |  |
|  | **renting/other** | 1.46 (1.05-2.04) |  | 1.45 (1.04-2.02) |  |
| **Smoking** | **never** | Ref. | <.001 | Ref. | <.001 |
|  | **ex-smoker** | 1.89 (0.98-3.62) |  | 1.89 (0.98-3.63) |  |
|  | **current smoker** | 3.02 (1.73-5.25) |  | 3.02 (1.73-5.26) |  |
| **Regular physical exercise** | **Yes** | Ref. |  | Ref. |  |
|  | **No** | 1.40 (0.87-2.26) |  | 1.41 (0.87-2.27) |  |
| **Eating alone** | **almost never** | Ref. | 0.15 | Ref. | 0.16 |
|  | **1-3 meals per week** | 1.50 (0.96-2.34) |  | 1.48 (0.95-2.32) |  |
|  | **4-6 meals per week** | 2.06 (1.10-3.86) |  | 2.07 (1.11-3.87) |  |
|  | **≥1 meal per day** | 1.32 (0.93-1.88) |  | 1.31 (0.91-1.86) |  |
| **Insomnia** | **No** | Ref. |  | Ref. |  |
|  | **Yes** | 2.34 (1.66-3.28) |  | 2.27 (1.61-3.21) |  |
| **History of chronic disease** | **0** |  |  | Ref. | 0.13 |
|  | **1** |  |  | 1.03 (0.72-1.47) |  |
|  | **≥2** |  |  | 1.66 (0.99-2.77) |  |
|  |  |  |  |  |  |
| ***AUC (95% CI)*** |  | *0.751 (0.717-0.785)* |  | *0.755 (0.721-0.789)* |  |
