# Supplementary material for: Gaps in universal health coverage in South Korea: Association with depression onset in a community cohort
Source: PLoS One. 2018 Jun 11;13(6):e0197679. doi: 10.1371/journal.pone.0197679 (PMC5995437; doi:10.1371/journal.pone.0197679)
Supplement: S3 Table — (DOCX) [file pone.0197679.s003.docx]

**S3 Table. Association between medical expense and depression onset at 4-year follow-up in different income groups (NHIS subjects only: N=3,286)**

|  |  | **Subgroup with monthly income below or at average (n=2,383)** | | **Subgroup with monthly income above average (n=891)** | |
| --- | --- | --- | --- | --- | --- |
|  |  | **Odds ratio (95% CI)** | **P-trend** | **Odds ratio (95% CI)** | **P-trend** |
| **Medical expenses per month (KRW)** | **almost none** | Ref. | 0.02 | Ref. | 0.48 |
|  | **<50,000** | 1.40 (0.90-2.18) |  | 1.59 (0.58-4.41) |  |
|  | **50,000-100,000** | 2.03 (1.21-3.43) |  | 1.28 (0.28-5.82) |  |
|  | **≥100,000** | 1.68 (0.94-3.03) |  | 1.84 (0.42-8.00) |  |
| **Age (yr)** |  | 1.04 (1.01-1.06) |  | 1.03 (0.96-1.11) |  |
| **Gender** | **Male** | Ref. |  | Ref. |  |
|  | **Female** | 2.90 (1.62-5.20) |  | 7.31 (1.59-33.61) |  |
| **Body mass index (kg/m^2^)** | **18.5-23** | Ref. | 0.02 | Ref. | 0.94 |
|  | **<18.5** | 2.81 (0.96-8.28) |  | N/A |  |
|  | **≥23** | 0.76 (0.53-1.08) |  | 0.89 (0.34-2.31) |  |
| **Education attainment** | **≥college** | Ref. | 0.004 | Ref. | 0.05 |
|  | **high school** | 1.41 (0.77-2.56) |  | 1.83 (0.62-5.41) |  |
|  | **≤middle school** | 2.15 (1.19-3.90) |  | 3.40 (0.97-11.93) |  |
| **Home ownership** | **home owner** | Ref. |  | Ref. |  |
|  | **renting/other** | 1.40 (0.98-1.99) |  | 2.34 (0.91-5.99) |  |
| **Smoking** | **never** | Ref. | <.001 | Ref. | 0.03 |
|  | **ex-smoker** | 1.85 (0.92-3.71) |  | 2.31 (0.37-14.57) |  |
|  | **current smoker** | 2.90 (1.60-5.24) |  | 5.19 (1.13-23.87) |  |
| **Regular physical exercise** | **Yes** | Ref. |  | Ref. |  |
|  | **No** | 1.45 (0.87-2.43) |  | 1.36 (0.39-4.72) |  |
| **Eating alone** | **almost never** | Ref. | 0.06 | Ref. | 0.43 |
|  | **1-3 meals per week** | 1.54 (0.95-2.51) |  | 1.38 (0.47-4.05) |  |
|  | **4-6 meals per week** | 2.54 (1.33-4.87) |  | N/A |  |
|  | **≥1 meal per day** | 1.46 (0.99-2.15) |  | 0.76 (0.30-1.89) |  |
| **Insomnia** | **No** | Ref. |  | Ref. |  |
|  | **Yes** | 2.34 (1.62-3.39) |  | 1.97 (0.72-5.38) |  |
| **History of chronic disease** | **0** | Ref. | 0.23 | Ref. | 0.22 |
|  | **1** | 1.06 (0.72-1.55) |  | 0.90 (0.31-2.59) |  |
|  | **≥2** | 1.5 (0.86-2.64) |  | 3.13 (0.88-11.18) |  |
